# Plant-Mycorrhizal Fungi Interactions in Phytoremediation of Geogenic Contaminated Soils

**DOI:** 10.3389/fmicb.2022.843415

**Published:** 2022-02-24

**Authors:** Ying Ma, Ankit, Jaya Tiwari, Kuldeep Bauddh

**Affiliations:** ^1^College of Resources and Environment, Southwest University, Chongqing, China; ^2^Department of Environmental Sciences, Central University of Jharkhand, Ranchi, India; ^3^Department of Community Medicine and School of Public Health, Postgraduate Institute of Medical Education and Research, Chandigarh, India

**Keywords:** phytoremediation, arbuscular mycorrhizal fungi, metal contaminated soils, metal transporters, genes

## Abstract

Soil contamination by geogenic contaminants (GCs) represents an imperative environmental problem. Various soil remediation methods have been successfully employed to ameliorate the health risks associated with GCs. Phytoremediation is considered as an eco-friendly and economical approach to revegetate GC-contaminated soils. However, it is a very slow process, as plants take a considerable amount of time to gain biomass. Also, the process is limited only to the depth and surface area of the root. Inoculation of arbuscular mycorrhizal fungi (AMF) with remediating plants has been found to accelerate the phytoremediation process by enhancing plant biomass and their metal accumulation potential while improving the soil physicochemical and biological characteristics. Progress in the field application is hindered by a lack of understanding of complex interactions between host plant and AMF that contribute to metal detoxification/(im)mobilization/accumulation/translocation. Thus, this review is an attempt to reveal the underlying mechanisms of plant-AMF interactions in phytoremediation.

## Introduction

As a result of rampant industrial activities, geogenic contaminants (GCs) have intruded in almost all spheres of the environment, including soil, water, air, and plants (Sandeep et al., 2019). Globally, the soils of >20 million hectares of land in 10 million sites are contaminated, and more than 50% of them are polluted with GCs (He et al., 2015). The root cause of this recurring problem of GC pollution seems to be the increased rate of industrialization, urbanization, mining, milling, fossil fuel burning, agrochemicals that release a wide range of GCs, and metalloids into the environment (Sandeep et al., 2019; Zhang et al., 2019). Leachates of municipal solid waste landfills in poor waste disposal systems contain elevated concentrations of GCs and metalloids, which are also responsible for contaminating soil soil-crop systems (Vongdala et al., 2019). The concentrations of GCs in soils may also be enhanced by applying inorganic and organic fertilizers, organic manure, pesticides, and herbicides (Dharma-Wardana, 2018; Reboredo et al., 2019). Several studies reported toxic metals accumulations in plant food samples harvested from contaminated soils, indicating that contaminated soils become the pathway of GCs to crops (Emurotu and Onianwa, 2017; Chaou et al., 2019; Kibet et al., 2019; Liang et al., 2019; Afonne and Ifediba, 2020). These released GCs are biomagnified in living beings of the higher trophic levels once they enter the food chain *via* the ingestion of food and vegetable.

Removal of GCs from the contaminated sites may be attained by various traditional techniques, such as detonation, incineration, soil excavation, soil washing, chemical precipitation, etc., which are very costly and adversely affect ecosystem functioning (Dermont et al., 2008). Recently, a widely used phytoremediation technique, the use of plants to extract, sequester, and detoxify pollutants, has been reported to be effective, non-intrusive, inexpensive, aesthetically pleasing, and socially accepted technology to remediate polluted soils (Kachout et al., 2009; Pandey and Bauddh, 2018). Soil amendment by using microorganisms, especially arbuscular mycorrhizal fungi (AMF) are efficient in accelerating the phytoremediation process (Ma et al., 2016). AMF inoculation is regarded as a promising tool in biotechnology for the sustainable remediation of hazardous contaminants (Schneider et al., 2017). Certain aspects in AMF associated phytoremediation, such as the response of plant and AMF species, the role of different soil parameters on their association, etc., needs to be well explored. Providing an in-depth literature review on the mechanisms responsible for plant-mycorrhizal fungi interactions in a lucid manner separates it from previous related work. Therefore, this paper expounds on the feasibility of a cost-effective and green method of AMF-assisted GC phytoremediation. Further, the mechanisms of action involved in plant-mycorrhizal fungi association for GC remediation from the contaminated sites have also been discussed.

### Methodology

The literature cited in this review ranged from 1904 to 2021. However, the majority of the articles targeted were from journal articles, book chapters, and books published between 2011 and 2021. The relevant literature surveyed were studied employing Google, Google Scholar, Web of Science, Research Gate, and Scopus using various keywords such as phytoremediation, arbuscular mycorrhizal fungi, metal contaminated soils, metal transporters, genes. Further, especially focused journals were Annual review of plant biology, Frontiers in Microbiology, Current Opinion in Toxicology, Journal of Plant Physiology, Plant Physiology, etc., were browsed for digging deeper into the relevant literature until 2021. Subsequently, we have examined the publication individually and eliminated the quotative and duplicate papers. Out of the total literature documents yielded, we have selected and referred to 168 articles. Out of which, total journal articles were 161 followed by six book chapters and one book. Around 51% of the cited documents were of the years 2011–2021. To the best of our knowledge, this article is an updated review article that focused and covered all dimensions of plant-mycorrhizal fungi interactions in metal phytoremediation.

## Establishment of Mutualistic Symbiosis

Soil can facilitate a conducive environment for interaction among diverse and highly complex microbial communities and is considered as a “safe haven” for them. Hiltner (1904) was the first soil biologist who defined the rhizosphere as a hyperactive “zone of contact” around the plant root system in the soil where microbes live and contribute to plant health. The findings of various studies suggested that rhizosphere processes are affected by exudates of plant roots and rhizosphere microorganisms (Kamilova et al., 2006; Kumar et al., 2007). Root exudates are involved in important functions, such as inducing plant defense response against pathogenic microorganisms (Abbott and Murphy, 2003) and providing a basis for chemotaxis to attract and repel microbial species and populations (Kumar et al., 2007), keeping the soil wet and moist, altering the chemical properties of the soils, mobilizing the nutrients, inhibiting the growth of competitor plants, and stabilizing soil aggregates around the roots. Root exudates mainly consist of carbon-based compounds (Bais et al., 2006), including low molecular weight compounds (e.g., amino acids, organic acids, sugars, phenolic, and several secondary metabolites), and high molecular weight compounds (e.g., mucilaginous substances and proteins; Badri and Vivanco, 2009).

The fungus-plant association fosters plant growth and boosts root development (Janeeshma and Puthur, 2020; Tiwari et al., 2020). Based on the basis of morphological characteristics, mycorrhiza is classified into five groups such as ecto-, ericoid, arbutoid, arbuscular, orchid, and monotropoid (Wang and Qiu, 2006). Among them, AMF is considered as most effective in promoting plant growth and development in the ecosystem by speeding up the processes of nutrient absorption. AMF starts symbiosis before they reach the host plant roots. During this pre-infection stage, plant roots release signal molecules (e.g., branching factors), which are responsible for the fast growth and branching of hyphae, followed by the differentiation of fungal adhesion structures. In reciprocation of branching factors, AMF may release signal molecules (e.g., Myc factors) that can induce both molecular and cellular responses and thus ensure successful AMF root colonization (Maillet et al., 2011). Positive results of this symbiosis are attributed to physiological changes of host plants, including hormonal equilibrium, transcriptional profile, primary, and secondary metabolism (López-Ráez et al., 2010).

## Performance of Mutualistic Symbiosis

**Figure 1:**
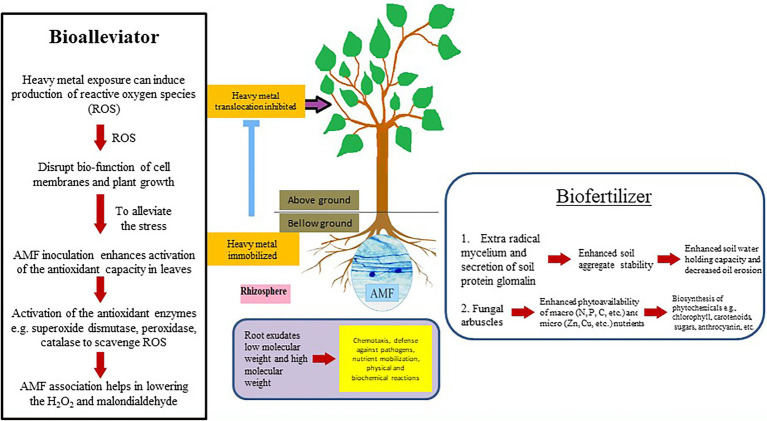
Plant-arbuscular mycorrhizal fungi (AMF) mutualistic performance in the rhizosphere.

Amongst several mutualistic symbioses, the arbuscular mycorrhizal symbiosis is considered as one of the significant determinants for plant health and soil fertility in terrestrial ecosystems (Jeffries et al., 2003). The fine hyphae that spread into the soil and absorb minerals more effectively than plant roots alone, and the presence of the fungi constantly decreases soil-borne fungi and nematode root attacks (Smith and Read, 2008). AMF may play important role in plant growth in metal contaminated soils (Hildebrandt et al., 2007) by acting as bioalleviator and/or biofertilizer (Figure 1). In addition, the large and dense mycelial network established by AMF improves the stability of soil particles through the excretion of glomalin (an insoluble and hydrophobic protein material) and soil proteins associated with glomalin, thus inhibiting disaggregation of soil organic carbon and water (Bedini et al., 2009; Hallett et al., 2009). AMF colonization can affect vegetative (Miller et al., 1987) and sexual reproduction of plants by influencing the number of inflorescences, production of seeds and fruits, and offspring vigor (Nuortila et al., 2004). These different attributes of AMF may contribute to protect endangered plants (Bothe et al., 2010). Following are some of the attributes that have been briefly discussed.

### Bioalleviator

The reactive oxygen species (ROS) formation accelerates under biotic and abiotic stresses (Hasanuzzaman et al., 2013; Bauddh and Singh, 2015; Jajic et al., 2015; Sachdev et al., 2021). ROS generation in plants has been reported as long-distance signals in response to various stresses (Mittler, 2017). To minimize the toxic effects of ROS, plants possess effective ROS-scavenging systems involving both enzymatic (e.g., ascorbate peroxidase and superoxide dismutase) and non-enzymatic (e.g., glutathione and ascorbic acid) ROS actions (Hasanuzzaman et al., 2013; Sachdev et al., 2021). Few researchers have reported that ROS generation occurs during early symbiotic interactions between AMF and host plant roots (Fester and Hause, 2005; Kiirika et al., 2014). To mitigate its potentially toxic effects, there must be a balance between ROS generation and scavenging. In plants, redox homeostasis, antioxidant signaling, and continuous production or removal of ROS at the cellular level are considered as stress signals (Jajic et al., 2015).

The plant-microbe symbiotic associations play a crucial role in alleviating biotic and abiotic stresses such as heat, salinity, drought, metals, and extreme temperatures (Goh et al., 2013; Schouteden et al., 2015). Studies on AMF mediated stress tolerance and increased growth of host plants have been the pivotal research on plant stress responses (Tahat and Sijam, 2012). Plant-AMF interactions can improve plant growth and health by controlling the generation and scavenging of ROS (e.g., H_2_O_2_, superoxide radicals, alkoxy radicals, singlet oxygen, perhydroxyl radicals, etc.) under biotic and abiotic stresses (Goh et al., 2013; Nath et al., 2016). For instance, a significantly higher amount of ROS is generated due to GC stress, therefore causing oxidative damage to the cellular structures of plants (Yang et al., 2015b). In response to such oxidative stress, plant-AMF associations can activate numerous antioxidant enzymes (e.g., thioredoxin, superoxide dismutase, glutathione peroxidases, and catalase) for scavenging the generated ROS to protect against cell damage (Hashem et al., 2018).

### Biofertilizer

AMF are considered natural biofertilizers, because they help the host plants to develop their root system for absorption of water and essential nutrients in exchange for photosynthetic products and to protect plants against harmful pathogens (Berruti et al., 2016; Gao et al., 2020; Basiru et al., 2021). It is well documented that, the AMF-pant association has countless benefits in terms of healthy soil conditions and increased crop productivity (Berruti et al., 2016). Therefore, AMF are considered as the most important biotic soil components, the impoverished or missing AMF can lead to a less efficient ecosystem functioning (Berruti et al., 2016). The roles of AMF as biofertilizer in several biochemical and physiological processes are as follows:

#### Phosphorus Acquisition

The phosphorus (P) absorption in the mutualistic relationship formed between the host plant and AMF offers significant advantages, such as, providing an efficient pathway through which P is retrieved from the soils and directly transferred to the roots. The exchange of nutrients between host plants and microorganisms is a regulated process facilitated by membrane transporting proteins such as, phosphate transport and the P-type H^+^-ATPase (Bucher, 2007; Basu et al., 2018).

#### Nitrogen Acquisition

Plant growth is often hampered by the loss of nitrogen from the soils. The mycorrhiza can facilitate nitrogen absorption from the soils to plants, and increase various types of nitrogen (Smith and Read, 2008; Veresoglou et al., 2011; Makarov, 2019). For instance, many studies demonstrated that plants associated with AMF have five times more affinity for NH_4_^+^ uptake from the soils (Pérez-Tienda et al., 2012). In addition, many mycorrhizal plants can facilitate nitrogen uptake from the rhizosphere soil through nitrate and ammonium transporters (Breuillin-Sessoms et al., 2015). Recently, few studies have also reported the increased content of ^15^N in host plants grown on AMF inoculated organic patches of soil (Hodge and Fitter, 2010; Tian et al., 2010; Nath et al., 2018). When the hyphae are supplied with nitrate and ammonium ions, the nitrates are absorbed by active transport coupled with a protonated-symport system, whereas, NH_4_^+^ is taken up through an antiport mechanism with an H^+^ efflux. If ammonium is the only source of N, its assimilation can lead to a deficient supply of carbon for the fungus because of its enhanced consumption in the roots (Basu et al., 2018).

#### Phytohormones

The fungal colonization develops in the host plant through a complex process that includes well-structured alterations at the morphological and genetic level, thus eventually leading to changes in series of signals (Morrison et al., 2015). Several studies have reported that AMF can produce phytohormones, e.g., auxins, cytokinins, and abscisic acid (ABA), which accelerated plant growth and development. Just as other plants, mycorrhizal fungi also follow the mevalonate pathway and use different precursors of ABA for their production (Nambara and Marion-Poll, 2005; Morrison et al., 2015). The role of ABA in the production and growth of mycelium has been documented in the literature (Ludwig-Müller, 2010; Spence and Bais, 2015; Xu et al., 2018). For instance, the exogenous application of ABA showed an insignificant increase in the growth of *Ceratocystis fimbriata*, while in *Magnaporthe oryzae*, ABA stimulated the production of appressoria and increased germination (Chanclud and Morel, 2016).

## Mechanisms Underlying Plant-AMF Interactions in Phytoremediation

Phytoremediation has been considered a more sustainable, cost-effective, and eco-friendly approach for the remediation of contaminated soils, due to its less expenditure and no unfavorable impact on soil fertility or structure (Jadia and Fulekar, 2009). However, phytoremediation cannot be performed alone by the plant itself, because plants and microorganisms in the rhizosphere always interact very closely so that ultimately leads to an enhanced activity associated with soil remediation (Compant et al., 2010). Use of hyperaccumulators associated with efficient endophytic or rhizosphere microbial communities has been proposed as a promising low-cost cleaning technique for the removal of metals from several contaminated sites (Karimi et al., 2011). In this context, AMF may be a good candidate because they reside inside the roots of a large number (approximately 80%) of terrestrial plants from bryophytes and tracheophytes (Smith and Read, 2008). AMF can form a mutual symbiotic association with most terrestrial plants establishing a direct physical link between plant roots and soils (Bothe et al., 2010).

AMF may promote plant metal extraction when metal concentrations are low in soils and also help plants to accumulate a major chunk of toxic metals in plant roots to prevent their transport to aerial parts when there is a high concentration of metals in soils. Singh et al. (2019) studied the impact of the inoculation of four species of AMF (e.g., *Rhizophagus fasciculatus*, *R. intraradices*, *Funneliformis mosseae*, and *Glomus aggregatum*) with *Zea mays* on the removal of Cr, Cd, Ni, and Pb from the tannery sludge. They discovered that all four AMF species enhanced metal accumulation in the roots but decreased shoot metal accumulation. The metal translocation factor was significantly lower as compared to the non-inoculated control plants. These discoveries are important evidence of the capability of AMF to enhance metal phytostabilization. Similarly, Yang et al. (2015a) evaluated the impact of two AMF *F. mosseae* and *R. intraradices* on plant growth-related parameters, Pb accumulation, photosynthesis, and antioxidant enzymatic activity in *Robinia pseudoacacia*. The increased biomass, photosynthetic pigment, gas exchange capacity, and various enzymatic activities in inoculated plants suggests that both AMF species were capable of protecting plants against cellular damage by eliminating ROS under Pb stress. The decreased Pb concentration in the leaves of AMF-inoculated *R. pseudoacica* indicates that these two AMF species have the potential to increase metal phytostabilization.

AMF root colonization helps in increasing the volume and surface area of available soil that in turn helps in better metal translocation from roots to shoots. Similarly, Zimmer et al. (2009) studied the impact of dual inoculation of ectomycorrhiza-associated bacteria (EMAB; *Sphingomonas* sp. and *Micrococcus luteus*) and ectomycorrhizal fungi (*Laccaria laccata* and *Hebeloma crustuliniforme*) on the growth and metal accumulation in *Salix viminalis* cultivated in metal contaminated soils. Total Zn and Cd accumulations in shoots were increased up to 53% post-inoculation with *H. crustuliniforme* in association with *M. luteus*, whereas up to 62% for *Sphingomonas* sp. They found that EMAB enhanced ectomycorrhiza formation, plant growth, and accumulation of Zn and Cd. The findings indicate that the bacterial community facilitates root colonization of plant growth-promoting ectomycorrhizal fungi, which may serve as a potential approach to increase the efficiency of phytoextraction. Moreover, a field study conducted by Wu et al. (2011) assessed the effect of AMF on Zn and Pb accumulation in *C. zizanioides* grown in mine tailings. They found that the P and N concentrations in plant aerial parts were remarkably higher in mycorrhizal plants as compared to non-AMF treatments. The inoculation of AMF also resulted in a decrease in Zn and Pb concentrations in roots.

The majority of studies available in the literature on AMF-assisted phytoremediation were performed in pot experiments using artificial GCs-polluted soils. Aged-contaminated soils are more complex than spiked soils, as they frequently contain different nature and concentrations of pollutants and their availability is generally lower than that in spiked soils. However, some studies have directly been performed at the contaminated site. It is a well-known fact that the nature of spiked soil used for pot experiments is different from those of naturally contaminated sites. Knowing about the behavior of plant species associated with AMF and the capability of such plants to grow in GC soils is imperative to phytoremediation (Schneider et al., 2016). It can thus be inferred that field studies depict the situation more closely. Therefore, there is a need to perform more and more field-based studies.

For instance, in a field study, a total of 23 species belonging to the genus *Acaulospora, Scutellospora*, *Racocetra*, *Glomus*, *Gigaspora*, and *Paraglomus* were identified in As contaminated areas in Brazil. The most frequently occurring species in all areas were *Paraglomus occultum*, *Acaulospora morrowiae*, and *Glomus clarum*. The relatively high presence of these species demonstrates their tolerance to excess As. In spite of the fact that contamination owing to As decreased AMF species richness, the presence of host plants has the tendency to make up for the reduction (Schneider et al., 2013a). In another field study, 39 species of AMF belonging to 10 genera were identified in Pb contaminated sites in Brazil. The *Acaulospora* and *Glomus* genera had a high occurrence in the rhizosphere and bulk soil. The highest concentration of Pb was found in root and shoot. AMF diversity seems to be correlated with the heterogeneity of area; AMF structure community was related to Pb concentration in soils, and the diversity of plants was significantly related to the diversity of AMF in soils with high Pb concentration. A clearer understanding of AMF communities in the presence of Pb stress may throw some more light on metal-fungal interactions in contaminated sites (Schneider et al., 2016). In a different field study, a total of six species of AMF belonging to two genera *Glomus* and *Scutellospora* were studied. The richness of AMF species was more in the non-contaminated site as compared to sites with contamination of metals. Results are suggestive of the fact that continuously exposing the plant and AMF to GC may result in the tolerant species that may be used for the purpose of phytoremediation (Khade and Adholeya, 2009). Metal transport followed by its distribution is imperative. Metal translocation from below ground to aerial parts is contingent upon the involved metals and plant species (Sarwar et al., 2017). The mobility of different metals differs even inside a plant. For instance, the mobility of Zn and Cd is higher than Pb and Cu. During transportation *via* plant, metals are largely bound to the root cell wall, which leads to enhanced metal concentration in the plant roots. Chelation of metals with the ligands (e.g., thiols, amino acids, and organic acids) facilitates the metals to transport from roots to shoots (Zacchini et al., 2009). Because of the high cation exchange capacity of xylem cells, the movement of metal is significantly retarded when metals are not chelated by ligands. There is an involvement of organic acids for Cd translocation in *Brassica juncea* (Salt et al., 1995), while histidine is involved in long-distance translocation of Ni in hyperaccumulator *Alyssum lesbiacum* (Solanki and Dhankhar, 2011). Since a larger number of GCs may be transported by forming organic compounds-metal complexes (Maser et al., 2001), various types of organic ligands secreted by AMF may alter the existing forms of metal distribution by combining with different metals present in plants, thereby assisting metal translocation from subsurface to aerial parts and hence improving the phytoextraction efficiency (Sheng et al., 2008). According to Ma et al. (2013), the inoculation of metal resistant plant growth-promoting bacterium *P. myrsinacearum* RC6b may effectively mobilize metals such as Pb, Cd, and Zn in soils and notably increased Cd and Zn accumulation in the shoots of *Sedum plumbizincicola*. De Maria et al. (2011) also observed that after inoculating rhizobacteria *Agromyces* sp. and *Streptomyces* sp., and fungus *Cadophora finlandica* with *Salix caprea*, the shoot concentration of Cd and Zn increased, denoting increased translocation of metals from roots to shoots.

There are a number of mechanisms through which plant-AMF interacts during the process of phytoremediation; some of them have been discussed below.

### AMF-Induced GC Detoxification

Accumulation of GCs in the plants is a critical problem in the environment, high mobility of GCs has made them an extended component of food chain that affects the health of humans. Vesicles present in mycorrhizal fungi are comparable to fungal vacuoles and they accumulate huge amount of GC in them (Dhalaria et al., 2020). Immobilization of GC occurs in the fungal hyphae residing in symbiotic association with plants that decrease their availability to plants by retaining the GCs in the cytoplasm or vacuole, cell wall by chelation, thereby reducing metal toxicity in the plants (Punamiya et al., 2010). Metal detoxification induced by AMF has been considered as the key mechanism to help plants to alleviate metal toxicity (Table 1). By using scanning electron microscope equipped with energy dispersive spectroscopy (SEM-EDS), extended X-ray absorption fine structure (EXAFS), linear combination fitting results of X-ray absorption near-edge spectroscopy (LC-XANES), it has been demonstrated that Cr may be immobilized by AMF *via* reduction of Cr (VI) to Cr (III), forming analogues of Cr (III)-phosphate, probably on the surface of fungi. Apart from this, it has also been unraveled that extra radical mycelium may take up Cr actively and transport it to mycorrhizal roots, but the majority of Cr is immobilized in fungal structures (Wu et al., 2015). Ultra-structural changes were observed in roots and leaves of *Leucaena leucocephala* through a scanning electron microscope (SEM), transmission electron microscope (TEM), and light microscopy (LS). Results revealed that plant tissues were colonized by AMF and damage was observed in all treatments of As (Schneider et al., 2013b).

**Table 1:** GC detoxification induced by AMF.

Possible mechanisms	References
Immobilizing geogenic contaminants (GCs) by secreting chelating substances, such as, siderophores (ferrichrome and ferricrocin) into the soil.	Ernst et al., 1992; Manoj et al., 2020
Metal-binding to several biopolymers present in cell walls such as glomalin and chitin. Glomalins are amphiphilic peptides that act as a surfactant.	Gonzalez-Chavez et al., 2004; Rillig and Mummey, 2006
Superficial immobilization of GCs in the plasma membrane upon crossing the cell wall.	Ernst et al., 1992
Intracellular chelation by metallothionein, organic acids, and amino acids.	Clemens, 2001
Arresting metals inside the vacuoles.	Gonzalez-Guerrero et al., 2008; Dhalaria et al., 2020
An exclusive mechanism of AMF involves metal transport with the help of fungal coenocytic hyphe.	Gonzalez-Chavez et al., 2002; Gupta et al., 2019
Membrane transporters present in arbuscules of AMF may transport metals to interfacial matrix and their incorporation in the plant.	Ebbs and Kochian, 1998
There is also a possibility that fungi may store metals in some assigned structures (such as vesicles, hyphe, etc.).	Ferrol et al., 2009

GCs may be immobilized in the fungal hyphae (Ouziad et al., 2004) that can fix GCs in the cell wall and store them in the vacuole or make a complex with other substances like glycoprotein-metal complex (Dhalaria et al., 2020) in the cytoplasm to decrease plants metal toxicity (Punamiya et al., 2010). AMF can enhance plant biomass by changing plant physiological and morphological properties (e.g., enhanced secondary metabolite levels, increased leaf area, increased seedling weight, etc.) under severely stressful conditions and uptake of important immovable nutrients (such as phosphorus, copper, and zinc; Miransari, 2017).

Increased plant biomass in rhizosphere soil is the primary cause of metal dilution in plant tissues (Audet, 2014). AMF may restrict Zn and Cd uptake in the cell wall of mental hyphae and cortical cells, thereby improving plant yield and health (Garg and Chandel, 2012). Metal detoxification mediated by *Rhizophagus phaseolina* in *Glycine max* was studied by Spagnoletti et al. (2016), where AMF boosted a defensive response by decreasing oxidative damage even in the presence of *M. phaseolina* and As.

Mycorrhizae may influence plant metal uptake from the rhizosphere and their translocation from the root zone to aerial parts (Li et al., 2015). The mycelium of several AMF has a high cation exchange capacity, and it helps in metal uptake (Takács and Vörös, 2003). For instance, Hammer et al. (2011) found an increase in the uptake of silicon in the hyphae and spores of *Rhizophagus irregularis* and its subsequent translocation to host roots. Cd toxicity and mobility can also be alleviated through AMF by enhancing the soil pH (Shen et al., 2006). AMF can restore Cd in the extraradical mycelium and bind Cd to glomalin (Janouškova and Pavlíková, 2010).

AMF colonization has been shown to reduce metal stress in a convincing way (Hall, 2002). For instance, AMF colonization considerably increased the glutamine synthetase activity, therefore enhancing Ni tolerance in *Berkheya coddii* (Sessitsch et al., 2013). To reduce metal toxicity, AMF resort to processes such as adsorption of GCs to the cell wall, immobilization of metallic compounds, chelation of GCs inside fungus, and precipitation of polyphosphate granules in soils (Meier et al., 2012; Wu et al., 2015). Janousková et al. (2006) reported that inoculation of *Glomus intraradices* with *Nicotiana tabacum* cultivated in Cd contaminated soil decreased Cd toxicity to the plants due to Cd immobilization in soil. A study conducted by Wu et al. (2015) investigated the mechanisms involved in Cr immobilization in *Daucus carota* inoculated with AMF and found that AMF can immobilize Cr *via* reduction of Cr(VI) to Cr(III) by forming Cr(III)–phosphate analogs.

### Molecular Regulation of Genes

Molecular regulation of genes plays a crucial role in accumulating GCs and fungal cell detoxification, subsequently leading to the prevention of translocation of these GCs toward the host plant (Emamverdian et al., 2015). Efflux of GCs is a strategy used by AMF to protect plants from metal toxicity (Latef et al., 2016; Shi et al., 2019). Several transcriptional genes take part in the efflux of GCs and the involved genes get activated by metal exposure (Dhalaria et al., 2020). To provide plant tolerance against Cd and Cu, *GmarMT1* that is a cDNA-encoding metallothionein-like functional polypeptide has been discovered from germinated *Gigaspora margarita* spores (Lanfranco et al., 2002). Also, GC exposure upregulates *GmarMT1* expression in the symbiotic mycelium (Lanfranco et al., 2002). *GintABC1* identified as a putative ATP-binding cassette (ABC) transporter from extra radical mycelium of *Glomus intraradices* is believed to be involved in Cd and Cu mitigation (González-Guerrero et al., 2010). A number of genes are responsible for maintaining cellular homeostasis against GCs, such as *GintABC1*, *GmarMT1*, *RintZn*, and *GrosMT1* (Azcón et al., 2013). To maintain the redox potential and safeguard the fungus from oxidative stress, *GmarMT1* codes for metallothioneins have been found in *G. margarita* BEG 34 (González-Guerrero et al., 2007). *GintABC1* assists in detoxifying Zn and Cu (González-Guerrero et al., 2007; Table 2). *RintZnT1* isolated from *Rhizophagus intraradices*, helps in vacuolar sequestration of Zn (Gonzalez-Guerrero et al., 2005). *GintGRX1*, the first characterized glomeromycotan glutaredoxin, is a multifunctional enzyme that expresses in response to oxidative stress (Benabdellah et al., 2009).

**Table 2:** The function of some receptor genes.

Receptor gene with their signaling component	Function	References
*BEG34/GintZnT1*	Enhanced transcription levels of putative Zn transporter gene and protection against Zn stress.	Gonzalez-Guerrero et al., 2005
*Sy167*	Alleviation of oxidative stress due to GCs.	Hildebrandt et al., 2007
*GintABC1*	Cd and Cu detoxification in the extra radical mycelium of *Glomus intraradices.*	Gonzalez-Guerrero et al., 2005
*MtCbf1* and *MtCbf2*	Root tissue reprogramming during the establishment of AM symbiosis.	Hogekamp et al., 2011
*Kinase SymRK*	Involved in transduction of signals to the cytoplasm after perception of signals from Nod and Myc factors.	Gherbi et al., 2008; Genre and Russo, 2016
*NUP 85/NUP133*	Involved in transporting macromolecules through nuclear envelope.	Parniske, 2008
*CYCLOPS*	Serves as phosphorylation target of calcium/calmodulin-dependent protein kinase (CCaMK) gene and is supposed to be the diverging point of common symbiosis (SYM) pathway.	Kistner et al., 2005
*CASTOR/POLLUX*	Specific channel of cations important for perinuclear Ca spiking right after reception of Myc or Nod factors.	Kistner et al., 2005
*CCaMK*	Calmodulin and Ca dependent protein kinase, which acts as a sensor of Ca and is supposed to be involved in phosphorylation of *CYCLOPS*.	Kistner et al., 2005

AMF resorts to several molecular mechanisms to protect them from GC stress. One of mechanisms is the upregulation of several transcriptional factors that activate glutathione-S-transferase and Zn transporter in intra- and extra- mycelia of AMF *Glomus intraradices* against metal stress (Hildebrandt et al., 2007). GC stress also leads to expression of numerous genes. These genes encoding proteins are involved in detoxification/tolerance against GCs (Rivera-Becerril et al., 2005).

Based on molecular understanding, scientists have reported an upregulation in metallothionein gene expression of *Gigaspora margarita* BEG34 in the symbiotic mycelia due to Cu (Lanfranco et al., 2002) and also an enhanced level of transcription of a putative transporter gene for Zn (*GintZnT1*) that belongs to cation diffusion facilitator family. These genes have been found in the G. intraradices mycelium under short and long-term Zn exposure indicating that this enzyme protects plants against Zn stress (Gonzalez-Guerrero et al., 2005). The role of some AMF in phytoremediation of some GCs (Bundschuh et al., 2017) has been discussed in Table 3.

**Table 3:** Role of AMF in phytoremediation of geogenic contaminated soils.

Plant	Types of mycorrhizae	GCs	Remarks	References
*Helianthus annus*	*Claroideoglomus claroideum* (BEG210)	Ni	AMF *Claroideoglomus claroideum* (BEG210) enhanced Ni accumulation in *H. annus* by 38%.	Ma et al., 2019
*Solanum nigrum*	*Rhizophagus irregularis*	Cd	*Rhizophagus irregularis* increased Cd accumulation in roots.	Wang et al., 2020
*Zea mays*	*Glomus aggregatum*	Pb, Cd, and Zn	AMF along with moderate amount of phosphorous may decrease GC uptake and increase plant growth.	Nafady and Elgharably, 2018
*Medicago sativa*	*Rhizophagus irregularis*	Cd and Ni	AMF inoculation enhanced the uptake of both metals.	Mnasri et al., 2017
*Taraxacum platypecidum*	*Rhizophagus irregularis*	Cr	Immobilized Cr in roots and prevents Cr phytotoxicity.	Wu et al., 2016
*Zea mays*	*Funneliformis mosseae* and *Diversispora spurcum*	Cd, Zn, Pb, and As	The transfer of GC was restricted by both fungi.	Zhan et al., 2018
*Solanum nigrum*	*Glomus intraradices*	Cd	Inoculation with AMF resulted in decreased Cd uptake in roots and shoots, thereby facilitating metal phytostabilization.	Khan et al., 2017
*Triticum aestivum*	*Rhizoglomus intraradices*	As	AMF inoculation assisted the host plant to ameliorate As-induced phosphorous deficiency and also strengthened thiol metabolism and antioxidant defence mechanism.	Sharma et al., 2017
*Cynodon dactylon*	*Funneliformis mosseae*	Sb	AMF inoculation inhibited Sb (V) to Sb (III) reduction, thereby decreasing Sb toxicity.	Wei et al., 2016
*Oryza sativa*	*Rhizophagus intraradices*	Cd	AMF decreased Cd uptake in *O. sativa* by altering the expression of Cd transporters.	Chen et al., 2019
*Zea mays*	*Glomus intraradices*	Hg	AMF increased Hg uptake in roots.	Debeljak et al., 2018
*Lactuca sativa*	*Funneliformis mosseae* and *Rhizophagus intraradices*	Zn	AMF inoculation at increased Zn concentrations AMF has the capability of decreasing Zn uptake.	Konieczny and Kowalska, 2017
*Cynodon dactylon*	*Diversispora spurcum*	Pb, Zn	AMF inoculation increased the uptake of Pb and Zn.	Zhan et al., 2019
*Sorghum bicolor*	*Claroideoglomus etunicat*	Mo	AMF inoculated plants accumulated up to four times higher Mo than non-mycorrhizal plants.	Shi et al., 2020

Metal-binding proteins called metallothioneins are generated in a diverse range of organisms when they are exposed to toxic metals (e.g., Cd, Zn, and Cu). Cu predominantly induces the production of metallothionein in non-AMF (Kumar et al., 2005). Cu-induced stress distinctly upregulates the metallothionein gene *BI451899* in extraradical mycelium of *G. intraradices*. However, a certain concentration of Zn can also upregulate the metallothionein gene, but such a response is not observed due to Cd. This upregulation establishes and supports the primary function of fungal metallothioneins in detoxifying Cu (Lanfranco et al., 2002).

### Metal Mobilization

Strong binding of metals to soil particles or precipitation results in insolubilization of the significant fraction of metals in soils ultimately causing their unavailability for plant uptake. Metal solubilization and mobility have been considered as critical factors that affect phytoextraction efficiency (Ma et al., 2013). In this regard, microbes that can mobilize metals may be used to amend the habitat of rhizosphere in soils affecting metal element speciation as well as mobility inside soil by way of biogeochemical cycling processes of GCs that primarily include protonation, chelation, and acidification (Ma et al., 2011; Rajkumar et al., 2012; Sessitsch et al., 2013).

### Protonation

AMF may also acidify their environment through exporting protons to replace GC cations at the site of sorption (Rajkumar et al., 2012). Extensive studies have been performed to characterize them using attenuated total reflection-Fourier transforms infrared (ATR-FTIR) spectroscopy and thereafter to understand the interaction between fungal cells, protons, and metal ions. Results suggest that the carboxylate moieties present on the bacterial surface play a vital role in the extracellular biosorption of Ni^2+^, which establishes a comparatively weaker bond with the metal ion.

### Chelation

Natural organic chelators are metal-binding compounds that comprise siderophores, organic acid anions, metallophores, and biosurfactants (Sessitsch et al., 2013). Both fungi and plants can release these compounds that scavenge metal ions from sorption sites (Gadd, 2004) and ROS (Leitenmaier and Küpper, 2013). Metal chelation through metal-binding peptides such as metallothioneins and phytochelatins (PC) may eliminate the harmful effect of free metallic ions, thereby facilitating metal uptake and their sequestration, followed by compartmentation, loading in xylem tissues, and finally their transport (Cai and Ma, 2002). Phytochelatins are the GC binding peptides that are produced by tripeptide glutathione and/or by an enzyme-catalyzed reaction through PC synthase (Solanki and Dhankhar, 2011). Metallothioneins may also be found in AMF and genes that encode numerous enzymes for PC synthesis may be activated in the root of mycorrhiza. These enzymes assist in enhancing photosynthesis in mycorrhizal plants subjected to stress caused by metals (Rivera-Becerril et al., 2005).

### Acidification

Soil pH is one of the most important factors that affect metal content and its bioavailability. For several metals (e.g., Cu and Zn), a rise in soil pH caused a fall in their mobility (Richards et al., 2000). Generally, soil pH is affected by the action of both microorganisms and plants. Rhizosphere gets acidified due to H^+^ ions excreted by roots that may displace GC cations adsorbed on soil particles. Root exudates may decrease the pH of the rhizosphere (Sheoran et al., 2011), causing increased metal mobility and bioavailability in soil solution (Kim et al., 2010).

### Metal Immobilization

Phytostabilization is GC immobilization in the plant root system by precipitation, reduction, and absorption without its accumulation in the shoot (Radziemska et al., 2017). There is an extensive root system for immobilizing metals in hyperaccumulators (Mendez and Maier, 2008). In addition to some common mechanisms of tolerance, increase biosynthesis of the cell wall, metal inactivation in the rhizosphere and its accumulation in roots are very specific to phytostabilizers (Janeeshma and Puthur, 2020). An association with AMF increases the properties of metal stabilization of plants (Zhang et al., 2019). For instance, the association of *Trifolium pratense* with mycorrhizae enhanced Zn retention in the roots, thereby preventing its translocation in the aerial plant parts (Chen et al., 2003).

The glomalins released by AMF enhance toxic metal immobilization. Metallothionein protein, released by some AMF, is also known to reduce the toxicity caused by GCs. Besides, the synthesis of a 90 kD heat shock protein and glutathione-S-transferase as a response to GC stress suggest that these proteins are involved in immobilizing GCs in the rhizosphere of *Lycopersicon esculentum* plant (Bano and Ashfaq, 2013). Glomalins are known to sequester several metals such as Zn, Pb, Fe, Cd, Cr, and Cu and decrease their bioavailability (Gil-Cardeza et al., 2014). Glomalins may extract Pb, Cd, and Cu from polluted soil.

Several GCs get immobilized because of the binding capacity of fungal hyphae to metals. As a result of this binding capacity, there is a decreased translocation of GCs to plant tissues (Wasserman et al., 1987). A slight increase in the mycorrhizosphere pH may also cause immobilization of some GCs (e.g., Zn) due to mycorrhizal association (Bano and Ashfaq, 2013). Inoculation of *Glomus* species resulted in reduced mobility of metals in *Zea mays* (Kaldorf et al., 1999). Other studies demonstrated a notable absorption of Zn in the mycelium of AMF by using different glomus species in association with *Lolium perenne* or *Trifolium* sp. (Joner et al., 2000).

Metal immobilized in fungal hyphae that are symbiotically associated with the plants decreases their availability to host plants by holding the metals in the cytoplasm, vacuole, or cell wall, thereby reducing metal toxicity in plants (Punamiya et al., 2010). They also immobilize the GCs in the root cortical region by binding with them and prevent the translocation of metals to shoot, thus preventing leaf tissue damage (Schubler, 2001). Some AMF may decrease plant metal uptake or its translocation factor by reducing metal bioavailability in soils through several processes such as alkalinization, precipitation, and complexation (Ma et al., 2016).

### Alkalinization

A few AMF exhibit the capability to adsorb metals by substratum alkalinization activity, hence affecting the stability of metals in soils (Büdel et al., 2004). The effect of alkalinization induced by AMF *via* release of OH^−^, may result in active uptake of nitrate by microbes and reduction in metal bioavailability in the rhizosphere by secreting glomalin (Giasson et al., 2008). AMF may act as a sink of metals to reduce the available and mobile metal cations in soils, resulting in the creation of a more conducive environment for plants growing in metal contaminated soils (Gohre and Paszkowski, 2006). Inoculation of *G. mosseae* and *G. caledonium* with *Lolium perenne* and *Sedum alfredi* notably reduced soil DTPA-extractable Cd by 21%–38% through the alkalinization process, hence facilitating in stabilization and extraction of Cd *in-situ* from Cd infected soils (Hu et al., 2013).

### Precipitation

Some plant-associated microorganisms can promote enzyme-catalyzed precipitation of toxic metals [e.g., chromium (Cr) and selenium] and radionuclides (e.g., technetium and uranium) *via* microbial reduction process, which is promising for phytoremediation of metal-polluted soils (Payne and DiChristina, 2006). Some studies suggest that fungi can protect the host plant from the inhibitory effects of an excess concentration of Cr^6+^ by reducing toxic and mobile Cr^6+^ to immobile and non-toxic Cr^3+^ in soils. Besides, some insoluble forms of minerals, metals, and radionuclides may also be immobilized either indirectly through bacterial oxidation of Fe (Zhou et al., 2013) or directly *via* enzymatic actions (such as microbial reduction process; Pagnanelli et al., 2010).

### Complexation

Extracellular polymeric substances (EPS) excreted by AMF are of immense importance, making a protective hindrance against the adverse effects of metal biosorption (Hou et al., 2013). The mechanisms involved in metal biosorption onto EPS include the complexation with negatively charged functional groups, precipitation, metal ion exchange, and adsorption (Zhang et al., 2006). In this regard, AMF may produce insoluble metal-absorbing glycoprotein named glomalin that decreases the metal mobility or sequesters them, which may be taken into account for metal stabilization in soils (Vodnik et al., 2008). In an *in-situ* field experiment, the glomalin-related soil protein was used to estimate AMF derived from glomalin in soils in sequestrating Pb and Cd (Wu et al., 2014).

## Conclusion

In this review, the interactions between plant and mycorrhizal fungi in metal phytoremediation were unraveled through (1) an in-depth establishment of mutualistic symbiosis; (2) gaining insight into the role of AMF in phytoremediation; and (3) understanding the mechanisms including alleviation of metal toxicity by AMF, plant-AMF signaling and perception, metal bioaccumulation of plant-AMF association, metal mobilization and immobilization, metal transport, and distribution, which could add to the existing application knowledge of phytoremediation technologies. Associations with mycorrhiza increase the available surface area for absorption beyond the zone of root hair that in turn increases the uptake of water and minerals. It results in the high production of biomass that is imperative for successful phytoremediation. This review combined all the existing information available on AMF in a coherent way for better understanding. The primary focus of upcoming research should be on (1) identification of new genes as well as gene products that are crucial in plant-mycorrhizae fungal interactions and (2) optimizing applied theory, including mobilization, immobilization, and perfecting the gene control mechanisms. The application of mycorrhizal techniques has fewer disadvantages and more advantages. Various factors such as redox potential, pH, inorganic and organic ligands (e.g., root exudates, fulvic acid, and humic acid) can regulate metal sorption or desorption and its bioavailability. The impact of the dynamics of these factors on phytostabilization, phytotransformation, or phytoextraction in association with AMF are still unclear and require more attention and detailed studies for additional application of phytoremediation processes. The review also advocates more and more field-based studies for further exploring the potential of AMF. Furthermore, applying it to practice, to enhance the utility and efficiency of mycorrhizal remediation of GCs are some practical problems that needs to be solved on an urgent basis.

## Author Contributions

All authors listed have made a substantial, direct, and intellectual contribution to the work and approved it for publication.

## Funding

This work is carried out at the College of Resources and Environment, Southwest University, supported by the Fundamental Research Funds for the Central Universities (no. SWU 020010), the Natural Science Foundation of Chongqing (no. cstc2021jcyj-msxmX0827) and Chongqing Returned Overseas Students’ Entrepreneurship and Innovation Support Program (no. cx2021001).

## Conflict of Interest

The authors declare that the research was conducted in the absence of any commercial or financial relationships that could be construed as a potential conflict of interest.
